# Drought stress triggers alterations of adaxial and abaxial stomatal development in basil leaves increasing water-use efficiency

**DOI:** 10.1093/hr/uhad075

**Published:** 2023-04-19

**Authors:** Elisa Driesen, Maurice De Proft, Wouter Saeys

**Affiliations:** Department of Biosystems, KU Leuven, Willem De Croylaan 42, 3001 Leuven, Belgium; Department of Biosystems, KU Leuven, Willem De Croylaan 42, 3001 Leuven, Belgium; Department of Biosystems, KU Leuven, Willem De Croylaan 42, 3001 Leuven, Belgium

## Abstract

The physiological control of stomatal opening by which plants adjust for water availability has been extensively researched. However, the impact of water availability on stomatal development has not received as much attention, especially for amphistomatic plants. Therefore, the acclimation of stomatal development in basil (*Ocimum basilicum* L.) leaves was investigated. Our results show that leaves developed under water-deficit conditions possess higher stomatal densities and decreased stomatal length for both the adaxial and abaxial leaf sides. Although the stomatal developmental reaction to water deficit was similar for the two leaf surfaces, it was proven that adaxial stomata are more sensitive to water stress than abaxial stomata, with more closed adaxial stomata under water-deficit conditions. Furthermore, plants with leaves containing smaller stomata at higher densities possessed a higher water use efficiency. Our findings highlight the importance of stomatal development as a tool for long-term acclimation to limit water loss, with minimal reduction in biomass production. This highlights the central role that stomata play in both the short (opening) and long-term (development) reaction of plants to water availability, making them key tools for efficient resource use and anticipation of future environmental changes.

## Introduction

To ensure that the available resources are used more efficiently, agriculture will need to adapt quickly to a changing world where the human population is rapidly growing and the climate is changing fast [1]. This demands the development of crop varieties able to sustain yields with less water input. The most sustainable and environment-friendly option for environmental stresses, including water shortage, is to complement precision irrigation with crops possessing a high water use efficiency. This can be achieved through either genetic engineering [2] or through cultivating stronger plants by the grower himself, with optimal anatomy for efficient transpiration and high yields. As previously reported by Driesen et al. (2021) for basil (*Ocimum basilicum* L.), water deficit reduces xylem vessel diameter whilst increasing vessel frequency in the stems, ensuring water conductivity with narrower hence stronger vessels [3].

Stomata regulate the gas exchange between the transpiring plant and the atmosphere. Stomatal development and opening are highly influenced by environmental cues [4]. By adjusting stomatal opening, plants optimize their CO_2_ uptake by unit water loss in the short term. Stomatal traits, such as stomatal density and size, have important consequences for the plants’ yield and survival [5]. So, in the long term, altering stomatal development may be the key to developing plants that acclimate to the dominating environmental conditions.

Stomata can be located on the abaxial (lower) leaf side (=hypostomatic) or the adaxial (upper) leaf side (=epistomatic), or on both sides (=amphistomatic). Bucher et al. (2017) concluded that more research needs to be conducted to unravel whether plant species change their stomatal characteristics, such as size and density, in parallel on either leaf surface or whether these changes can occur independently. This is especially relevant because of the reported differences between adaxial and abaxial stomata, both anatomically and in their response to environmental changes. Firstly, adaxial leaf surfaces usually have lower stomatal density than the abaxial leaf surface [6]. Secondly, abaxial and adaxial stomata are reported to behave independently in response to environmental cues [7–9]. The reaction of stomatal opening and development to environmental cues has been investigated for different CO_2_ levels [10], light [7, 11], and water status [12, 13]. The impact of drought on stomatal density and size is ecologically and economically important, especially concerning the efficient use of resources. Xu and Zhou (2008) found that the size of the abaxial stomata in perennial grass (*Leymus chinensis*), a monocot plant, decreased with water deficit while the stomatal density increased (up to a certain point of moderate water deficit). However, Hamanishi et al. (2012) found the opposite in two genotypes of *Populus balsamifera* (dicot plants), with leaves developed under water-deficit conditions showing lower stomatal indices compared to leaves developed under well-watered conditions.

The main reasons for stomatal size and density breeding are (*i*) maximizing productivity by increasing assimilation rates, and (*ii*) improving drought tolerance [14]. Nevertheless, diverse other reasons are notable such as disease resistance, tolerance against atmospheric pollutants, evaporative cooling, increasing water use efficiency, and high-temperature tolerance [14, 15]. Herbaceous plants have been hypothesized to favor amphistomaty to help them optimally balance light acquisition with gas exchange under high light conditions [16, 17]. Mott et al. (1982) had already hypothesized that a possible advantage of the formation of adaxial stomata is the addition of an additional boundary layer conductance to the overall diffusion pathway, parallel with the abaxial surface. This raises the maximum leaf conductance for CO_2,_ which is advantageous in environments with high sunlight, making the intercellular CO_2_ concentration the only significant factor limiting carbon assimilation [18–20]. In herbaceous plants, where the abaxial and adaxial pathways are already short because of the thin leaves, amphistomaty enhances the leaf-atmosphere gas exchange capacity, as they effectively halve the leaf boundary layer resistance by using both leaf surfaces for gas exchange [21]. However, Terashima *et al.* (2001) suggest that annual herbs are amphistomatous as they need to attain considerable mesophyll surface area for efficient light interception by having thick leaves. They hypothesize that annual herbs need to expand their leaves quickly, thus having leaves with big cells is advantageous for quick leaf expansion. Similarly, Mott *et al.* (1982) noticed a correlation between leaf thickness and amphistomaty for various common dicotyledonous species in Arizona, California, and Mexico. However, it should be noted that there is a great overlap between the range in leaf thickness of amphistomatous and hypostomatous leaves. They suggest that although there is a correlation between leaf thickness and amphistomaty, this should be considered a secondary correlation [18, 22]. It should be noted that amphistomaty also increases transpiration by the formation of this additional boundary layer conductance for water transport, which represents a cost of amphistomaty compared to hypostomaty [23]. Additionally, amphistomatic leaves can simultaneously supply water to both leaf surfaces using a single vascular network, which minimizes building costs [24]. The impact of stomatal distribution on stomatal conductance is thus an important factor regarding amphistomaty [20, 25]. Increased leaf CO_2_ diffusion conductance and enhanced photosynthesis were noted when shifting from hypostomatous to amphistomatous leaves [20, 25].

The anatomical form of the leaf on each leaf side may affect leaf stomatal conductance, as amphistomatic leaves are not morphologically uniform. The dorsiventral leaves have an altered differentiation of mesophyll cells into palisade and spongy mesophyll tissue, with densely packed palisade parenchyma near the upper epidermis (small intercellular spaces) and loosely arranged spongy parenchyma near the lower epidermis (large intercellular spaces). Other possible benefits and costs of dorsiventral amphistomatous leaves can be found in the review of Drake et al. (2019), where the possibility of condensation around the transition between palisade and spongy mesophyll in the leaves is also discussed [21, 26]. Nevertheless, the occurrence of amphistomaty suggests an evolutionary trend, where the higher photosynthesis rates may have acted as a selective force favoring the development of amphistomatic plants [17, 18, 21, 27].

As few reports were found dealing with the response of adaxial stomata to environmental cues such as drought, this study evaluated the effect of water-deficit conditions and well-watered conditions on the development of the stomata on both leaf surfaces and all leaf levels of two cultivars of basil grown in growth chamber conditions. It was also investigated if newly developing leaves acclimate their stomatal development quickly dependent on the current soil water availability. To this end, the water availability was switched during cultivation, and the stomatal development and conductance of newly formed leaves were analyzed. The main aims of this study were (*i)* to clarify the response for abaxial and adaxial stomatal development in basil leaves to water availability, and (*ii*) to determine if differences in stomatal development exist for the two surfaces and hence decide if the observed reactions are adaptive. To this end, we investigated the impact of drought on stomatal development during leaf formation by testing the hypothesis that water-deficit conditions increase stomatal density and decrease the stomatal size for both leaf sides, hence enhancing drought tolerance, water-use efficiency, and stomatal conductance. More specifically, we set out to test the hypothesis that leaves induce an appropriate stomatal developmental response dependent on their current soil moisture content conditions.

**Figure 1:**
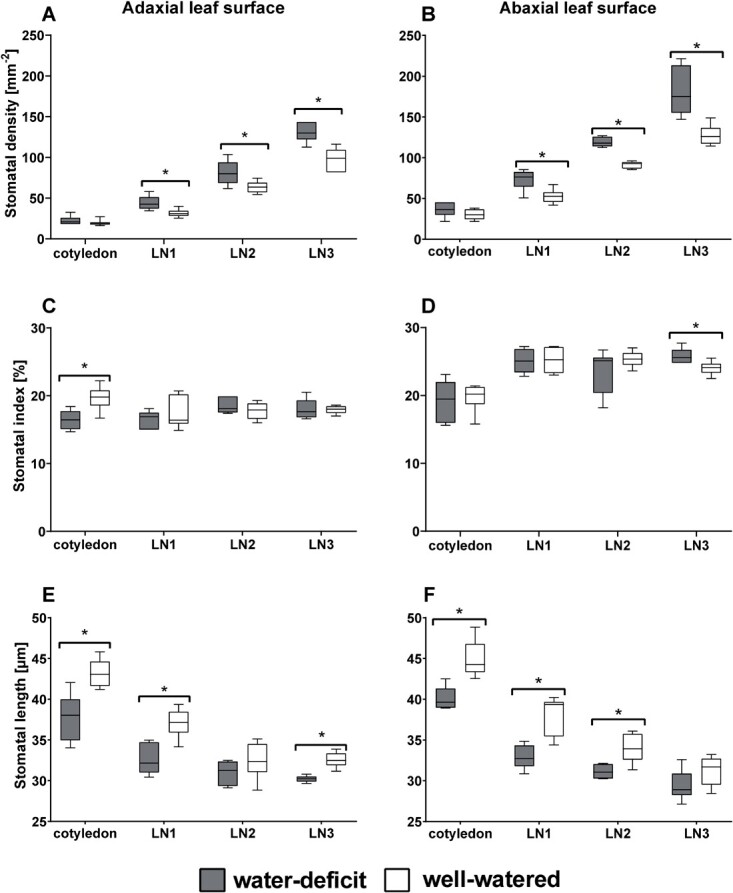
Box plots of the variation in stomatal density SD [mm^−2^] of basil plants at different leaf levels for adaxial (A) and abaxial (B) leaf surfaces, stomatal index SI [%] for adaxial (C) and abaxial (D) leaf surfaces, and stomatal length SL of basil plants; both adaxially (E) and abaxially (F) at 35 DAS. Asterisks mark significant differences between well-watered and water-deficit treated plants, at the different leaf levels (p < 0.05, n = 6) using an ANOVA test.

**Figure 2:**
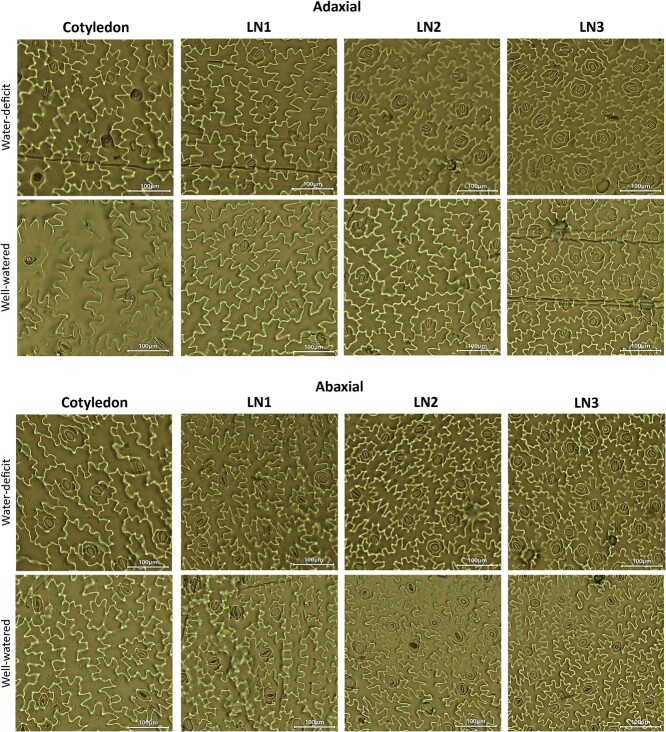
Representative micrographs of the stomata located on the leaves of all leaf levels, cultivated under water-deficit (left) and well-watered (right) conditions at 35 DAS. The adaxial (upper) and abaxial (lower) stomata are displayed. The scale bar represents 100 μm

**Figure 3:**
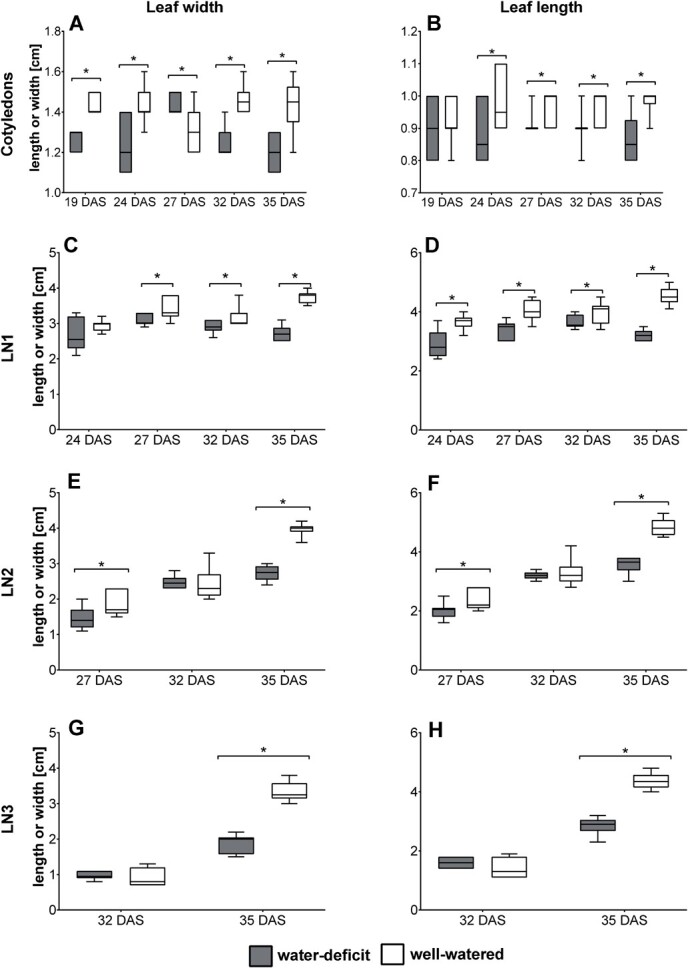
Box plots for leaf width [cm] (left) and leaf length [cm] (right) of the cotyledons (A, B), leaf number 1 (C, D), 2 (E, F), and 3 (G, H) at 35 DAS during cultivation under water-deficit and well-watered conditions. Asterisks indicate significant differences (p < 0.05, *n* = 10) using an ANOVA test.

## Results

### Drought stress leads to a larger number of smaller stomata on both sides of the leaf

Cultivation at water-deficit conditions gave similar responses in the stomatal anatomy on the adaxial and abaxial surfaces of basil leaves: the stomatal density *SD* increased (Figures 1.A and 1.B) while their length *SL* decreased (Figures 1.E and 1.F). The stomatal index *SI* stayed constant (Figure 1.C and 1.D). Figure 2 displays representative micrographs of the adaxial and abaxial stomata located on the leaves of all leaf levels at 35 DAS, cultivated under water-deficit and well-watered conditions. This indicates that basil plants acclimate their stomatal development according to the water availability during cultivation (Figure 1). On the adaxial leaf surface, the decrease in *SL* due to the water-deficit treatment was significant at the cotyledon level (−11%), at LN1 (−12%) and, at LN3 (−9%) (Figure 1.E). On the abaxial leaf surface, *SL* decreased significantly at the cotyledon level (−10%), at LN1 (−17%), and LN2 (−12%) (Figure 1.F). While the stomatal length *SL* decreased with water deficit, its density *SD* significantly increased both adaxially and abaxially (Figure 1.A and 1.B). The number of stomata was always higher on the abaxial leaf surface than on the adaxial leaf surface for both treatments. These changes (in percentages) in stomatal numbers due to different water availability were similar on the adaxial side to those on the abaxial side for each leaf level. As indicated in Figure 1.A, significant increases in stomatal density *SD* on the adaxial leaf surface due to water deficit conditions were observed for LN1 (+42%), LN2 (+27%), and LN3 (+35%). Similarly, significant increases of 38% (LN1), 31% (LN2), and 41% (LN3) were observed on the abaxial leaf surface (Figure 1.B). The stomatal index *SI* values were not significantly different between the water-deficit and well-watered treatment for both leaf surfaces, with the exception of the cotyledons (adaxial leaf side) and LN3 (abaxial leaf side) (Figures 1.C and 1.D).

Despite the different leaf levels and water availability, there was a strong negative, non-linear relation between *SD* and *SL*, on either one or both leaf surfaces. This functional trade-off between *SD* and *SL* can be described by a log-normal model: log(*SD*) = −4.9854 x log(*SL*) + 9.407 with *R*^2^ = 0.65 for water-deficit conditions, and log(*SD*) = −3.9236 x log(*SL*) + 7.8495 with *R*^2^ = 0.70 for well-watered conditions. The characteristics of the well-watered versus water-deficit treatments can easily be distinguished, translating to a maximal *SL* of 76.91 μm and 100.14 μm for stomata cultivated under water-deficit or well-watered conditions, respectively.

**Figure 4:**
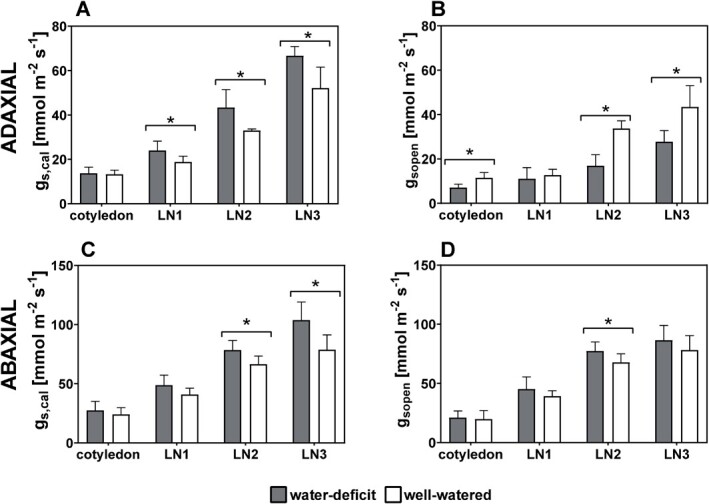
The effect of stomatal developmental acclimation of basil plants grown under water-deficit and well-watered conditions on the potential stomatal conductance (g_s,cal_, in mol m^−2^ s^−1^) both adaxially (**A**) and abaxially (**C)** and on the stomatal conductivity (g_s,open_ in mol m^−2^ s^−1^), based on the number of open stomata (SD_open_), both adaxially (**B**) and abaxially (**D**). Asterisks mark significant differences between well-watered and water-deficit treated plants, at the different leaf levels (p < 0.05, n = 6) using an ANOVA test.

The stomatal density and size also significantly differ between leaf numbers, with older leaves having the lowest density with the largest stomatal lengths (Figures 1 and 2). This shows the impact of cell expansion dependent on leaf development, aging, water content, and the positioning of the leaves. With epidermal cell areas ranging from 2877.1.3 ± 529.4 μm^2^ (LN3, pF1.95) to 6942.2.7 ± 729.2 μm^2^ (LN1, pF1.15), the mean response is a 33% and 44% reduction in epidermal area from LN1 to LN2 (water-deficit and well-watered treatments resp.) and a 44% and 30% reduction in epidermal area from LN2 to LN3. Between the different irrigation treatments, there were significant differences for leaf levels of LN1 (abaxial) and LN2 (abaxial and adaxial) in epidermal cell areas, with the well-watered treatment having the largest epidermal cell areas on each leaf level, indicating the cell expansion possibilities under well-watered conditions (data not shown). In contrast to the epidermal cell area, the epidermal cell number decreased by about 38 ± 6% between each leaf level. Between treatments and within leaf level, the epidermal cells were about 26 ± 3% less abundant in the well-watered treatments compared to the water-deficit treatment.

Leaf width and leaf length significantly differed between the two treatments for the different leaf levels (Figure 3), with leaves from the well-watered treatment being significantly larger. Leaf width increased between 7–14% (cotyledons), 9–29% (LN1), 21–33% (LN2), and 42% (LN3, 35 DAS). Leaf length showed increases of 10% (cotyledons), between 8–29% (LN1), 17–25% (LN2), and 34% (LN3, 35 DAS). Increased leaf size under well-watered conditions, or decreased leaf size under water-deficit conditions, has been reported by many researchers [33–38]. As the stomatal index does not significantly differ between the two treatments (Figure 1), and the epidermal cell area increases under well-watered conditions, the increased leaf area is likely the result of cell expansion under well-watered conditions.

As illustrated in Figure 4, the stomatal acclimation to drought, translating in an increase of density *SD* and decrease of length *SL* had an impact on *g*_s,cal_, the potential stomatal conductance based on the stomatal anatomy calculated with Eqn. [1]. Plants cultivated under water-deficit conditions have the potential to transpire more compared to well-watered treatments, thanks to acclimation of their stomata to increase the calculated potential stomatal conductance *g*_s,cal_ (Figure 4.A and 4.C). The *g*_s,cal_ values for the adaxial leaf side under water-deficit conditions of 24.0 ± 4.3 (LN1), 43.4.2 ± 8.0 (LN2) and 66.7 ± 4.2 mmol m^−2^ s^−1^ (LN3) are all significantly higher compared to the well-watered treatment of 18.8 ± 2.6 (LN1), 35.5 ± 6.0 (LN2) and 52.2 ± 9.4 mmol m^−2^ s^−1^ (LN3) (Figure 4.A). On the abaxial leaf side, these significant differences in *g*_s,cal_ were also present for LN2 and LN3: 78.4 ± 8.1 versus 66.4 ± 6.9 mmol m^−2^ s^−1^ (LN2) and 103.7 ± 15.4 versus 78.8 ± 12.5 mmol m^−2^ s^−1^ (LN3) for water-deficit and well-watered treatments, respectively (Figure 4.C). Copolovici *et al.* (2021) reported similar values for basil plants [39].

Although the plants grown under the water-deficit treatment have the potential to transpire more based on their stomatal anatomical features, plants in water-deficit will not have all their stomata open. Therefore, the number of open stomata (*SD*_open_) was counted and *g*_s,open_ was calculated using the same equation for *g*_s,cal_ (Eqn. [1]), but substituting stomatal density *SD* with *SD*_open_ (Figure 4.B and 4.D). Adaxial stomata are more sensitive to drought stress compared to stomata on the abaxial leaf side, as more stomata on the upper leaf side closed due to water-deficit conditions compared to the lower leaf side. This translates into a significant reduction of 49% (cotyledons), 60% (LN1), 61% (LN2), and 59% (LN3) in the calculated *g*_s,open_ (adaxial) compared to *g*_s,cal_ under water-deficit conditions (Figure 4.B and 4.A). In comparison, the reduction from *g*_s,cal_ to *g*_s,open_ (adaxial) under well-watered conditions was much smaller with significant reductions of 15% (cotyledons), 33% (LN1), and 15% (LN2). This remarkable decrease in stomatal conductance when taking the open stomata into account was not significant for the abaxial leaf side.

### Water availability influences the stomatal development of the still-growing plants

The observed significant differences in stomatal density *SD* and length *SL* between plants cultivated in well-watered and water-deficit conditions at 35 DAS (Figure 1) already manifested themselves earlier during the developmental stage. At 19, 24, 27, 32, and 35 DAS, epidermal peels were taken for six leaves per treatment, both abaxial and adaxial for all leaf numbers present at that developmental stage. Already at 27 DAS, the water-deficit treatment showed significantly higher *SD* for the adaxial surface of LN1 and LN2 and the abaxial surface of the cotyledons and LN 1 (Table 1). From this, it can be concluded that already in the early stages of the cultivation period the number of stomata is strongly regulated by the soil moisture content.

**Figure 5:**
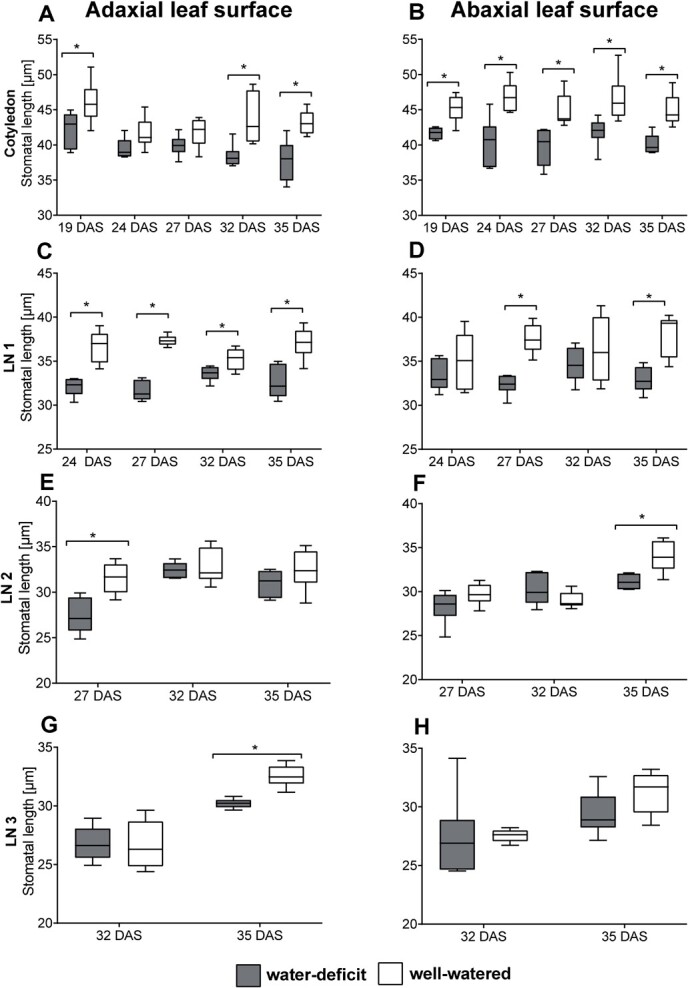
Box plots for the stomatal length *SL* [μm] at the adaxial (left) and abaxial (right) leaf side of the cotyledons **(A, B)**, leaf number 1 **(C, D)**, 2 **(E, F),** and 3 **(G, H)** at different stages (DAS, *days after sowing*) during cultivation under water-deficit and well-watered conditions. Asterisks indicate significant differences (p < 0.05, *n* = 6) using an ANOVA test.

Moreover, this acclimation plant response of stomatal development among adaxial and abaxial leaf sides could also clearly be seen in stomatal length *SL* (Figure 5). Starting from 19 DAS, significant differences in *SL* both adaxially and abaxially were observed in the cotyledons and became significant later on in the other leaf numbers. The water-deficit treatment showed significantly smaller stomata compared to the well-watered treatment. This was observed on both leaf sides and for all leaf numbers. Plants grown under water-deficit conditions acclimate to this condition by forming smaller stomata.

**Table 1:** Effect of soil moisture content (well-watered or pF1.15, and water-deficit or pF1.95) on the stomatal densities *SD* [mm^−*2*^] of abaxial and adaxial leaf sides from the cotyledons to the uppermost leaf (LN3) at 27, 32 and 35 days after sowing (DAS) of basil plants. The data shows the mean values ± standard deviation (*n* = 6). The different letters represent the statistical differences at p < 0.05 for each DAS and leaf number, analyzed using an ANOVA test

		27 DAS		32 DAS		35 DAS	
LS	LN	Water-deficit	Well-watered	Water-deficit	Well-watered	Water-deficit	Well-watered
Abaxial	Cot.	19 ± 2	20 ± 3	21 ± 5	16 ± 5	22 ± 5	19 ± 4
	LN1	48 ± 5 (a)	40 ± 4 (b)	44 ± 9	46 ± 13	44 ± 8 (a)	31 ± 5 (b)
	LN2	138 ± 24 (a)	108 ± 17 (b)	83 ± 8 (b)	102 ± 14 (a)	81 ± 15 (a)	64 ± 7 (b)
	LN3			175 ± 26	190 ± 43	131 ± 12 (a)	97 ± 14 (b)
Abaxial	Cot.	40 ± 6 (a)	32 ± 5 (b)	33 ± 3	31 ± 5	36 ± 9	30 ± 6
	LN1	73 ± 9 (a)	53 ± 2 (b)	82 ± 4 (a)	69 ± 10 (b)	73 ± 12 (a)	53 ± 9 (b)
	LN2	143 ± 34	157 ± 24	142 ± 13	138 ± 20	119 ± 6 (a)	91 ± 4 (b)
	LN3			195 ± 50	190 ± 35	181 ± 30 (a)	128 ± 12 (b)

### Acclimation of stomatal densities and sizes of newly developing leaves depends on available water

Acclimation of the leaf stomatal anatomy of newly developing leaves to water deficit was investigated using two different basil cultivars: cv. Marian and cv. Gustosa. Leaves of plants grown for 42 DAS at the same matric potential (“No VWC change”) were compared to leaves of plants that changed after 35 DAS to another matric potential (“After VWC change”) (Table 2).

Compared to the plants that stayed in water-deficit conditions, the stomatal cells on the newly formed leaves of the plants that were transferred from water-deficit conditions to well-watered conditions showed significantly lower *SD* (Table 2), decreasing from 131 ± 12 mm^−2^ to 88 ± 17 mm^−2^ (adaxially, Marian) and 181 ± 30 mm^−2^ to 119 ± 8 mm^−2^ (abaxially, Marian). The opposite was found in newly formed leaves in plants transferred from well-watered conditions to water-deficit conditions. These newly formed leaves showed an increase in *SD* of 44% adaxially and 54% abaxially (Marian) while decreasing their *SL* significantly with 8% adaxially (Marian) and 17% abaxially (Gustosa). This indicates that the leaf emerged fully acclimated to the current water availability status.

**Figure 6:**
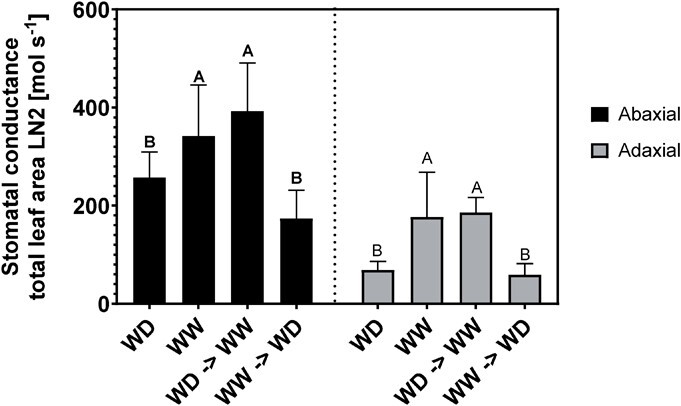
acclimation of stomatal conductance (mol s^−1^) of the total leaf area of LN2 (abaxial and adaxial) to soil moisture content change during the last week of the cultivation period. The cultivation containers were either kept at their initial pF values (WW (well-watered) or WD (water-deficit) for the 5-week cultivation period) or changed to their opposite value: WW (4 weeks) – WD (1 week) or WD (4 weeks) – WW (1 week). Data show the mean values (*n* = 5) ± SD in each case. The connecting letters report shows the significant difference between the different treatments for the abaxial and adaxial leaf surface separately (p < 0.05) using the Wilcoxon Rank Sum test.

To test whether the stomatal anatomy is at the basis of this manipulation of evaporative water loss, the stomatal conductance of LN2 was measured at the end of the cultivation period, as well as microscopic peels were made of that leaf area to investigate the stomatal anatomy in relation to the stomatal conductance measurements. A linear relationship was found between the measured stomatal conductance *g_s_* and the calculated potential stomatal conductance *g_s,cal_* (R^2^ = 0.51). Figure 6 displays the acclimation of the measured stomatal conductance *g_s_* for the total leaf area of LN2 to the changes in soil moisture content. The *g_s_* of the abaxial and adaxial leaf side was significantly different between the water-deficit and well-watered treatments, with the values for the well-watered treatment being the highest. This indicates that *g_s_* was acclimated to cope with the change in VWC: when plants changed from well-watered to water-deficit conditions, their *g_s_* decreased both adaxially and abaxially. The stomatal conductance *g_s_* decreased with 49% (abaxial leaf side) and 67% (adaxial leaf side) when switching from well-watered to water-deficit conditions, showing a higher reduction in stomatal conductance for adaxial stomata, congruent with the data reported in Figure 4.

When comparing the stomatal conductance *g_s_* measurements of LN2 (Figure 6) to the calculated potential stomatal conductance *g_s,cal_* of LN2 (Figure 4), values of similar orders of magnitude were found, expressed per leaf area [mol s^−1^], using 9.5 cm^2^ and 8 cm^2^ as average leaf area for LN2 of well-watered and water-deficit treatments, respectively. The average *g_s,cal_* values of LN2 were 627.3 ± 64.6 (WD, abaxial), 631.0 ± 65.8 (WW, abaxial), 346.9 ± 64.4 (WD, adaxial) and 336.9 ± 56.8 (WW, adaxial). These values are higher than the measured values as expected, especially for the water-deficit treatment (Figure 6), but still in a similar range.

### Stomatal characteristics and their relationship with WUE

**Figure 7:**
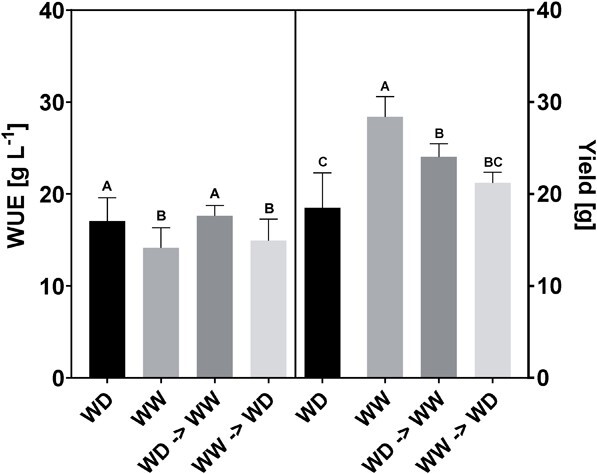
WUE [g L^−1^] (left) and yield [g leaves and stems] (right) of basil plants (cv. Gustosa) cultivated under water-deficit (WD) and well-watered (WW) conditions until 42 DAS, compared to the WUE and yield of basil plants cultivated at the same matric potential for 35 DAS and then switched to opposite matric potential until 42 DAS: water-deficit switched to well-watered (WD ➔ WW) or well-watered to water-deficit (WW ➔ WD). Data represent average ± standard deviation. Significant differences according to a Tukey HSD test are indicated by the significant letters report (p < 0.05, n = 6).

The water use efficiency (WUE, in g fresh weight L^−1^) was significantly higher in the water-deficit treatments at 18.79 ± 5.27 and 17.07 ± 2.51 g L^−1^ in the experiments with cv. Marian and cv. Gustosa (Figure 7) respectively, compared to the corresponding WUE of the well-watered treatments at 13.55 ± 1.01 and 14.16 ± 2.18 g L^−1^. Plants exposed to drier conditions displayed a lower biomass production compared to plants exposed to well-watered conditions, 18.49 ± 3.80 g versus 28.38 ± 2.20 g fresh weight of stems and leaves. However, exposing plants to water-deficit conditions and then switching to well-watered conditions increased biomass production significantly compared to the full water-deficit treatment, while maintaining a high WUE. Plants (cv. Gustosa) that were cultivated for 4 weeks at pF1.95 and switched to pF1.15 in the last cultivation week, displayed a final biomass of 24.03 ± 1.44 g fresh weight, which was significantly higher than the biomass production for the full water-deficit treatment (18.49 ± 3.80 g). These plants also displayed a WUE of 17.62 ± 1.13 g L^−1^, similar to the water-deficit treatment and significantly higher than the well-watered treatment (Figure 7). The opposite phenomenon occurred in plants first exposed to well-watered conditions (4 weeks) and then transferred to water-deficit conditions (1 week). Their fresh weight per plant container was 21.20 ± 1.16 g at the end of the cultivation period, which is significantly lower than for the well-watered treatment and similar to the values for the water-deficit treatment. Similarly, their WUE of 14.91 ± 2.36 g L^−1^ is significantly smaller compared to the water-deficit treatment and similar to the well-watered treatment.

**Table 2:** comparison of acclimation effects of the *SD* and *SL* on the adaxial and abaxial leaf surface of newly formed, fully expanded leaves of basil plants. Samples (*n* = 6) were taken from LN3 at 42 DAS (days after sowing). Plants that were cultivated at a matric potential of pF1.95 (water-deficit) and pF1.15 (well-watered) for 42 DAS (“No VWC change”) are compared with plants that were cultivated at the same matric potential for 35 DAS and then switched to the opposite matric potential until 42 DAS (“After VWC change”). This experiment was done twice, once with cv. Marian and once with cv. Gustosa. Data represent average ± standard deviation. Significant differences according to a Wilcoxon multiple comparison test are indicated with an asterisk (p < 0.05)

			No VWC change	After VWC change	%	Significance
**Stomatal density [mm** ^**−2**^ **]**						
water-deficit ➔ well-watered	Adaxial	Marian	131 ± 12	88 ± 17	−30	*
		Gustosa	107 ± 16	86 ± 6	−20	ns
	Abaxial	Marian	181 ± 30	119 ± 8	−32	*
		Gustosa	124 ± 11	78 ± 19	−37	ns
well-watered ➔ water-deficit	Adaxial	Marian	97 ± 14	141 ± 4	+44	*
		Gustosa	115 ± 3	132 ± 4	+15	ns
	Abaxial	Marian	128 ± 12	191 ± 11	+54	*
		Gustosa	103 ± 26	149 ± 17	+44	ns
**Stomatal length [μm]**						
water-deficit ➔ well-watered	Adaxial	Marian	30.3 ± 0.4	32.2 ± 1.4	+6	ns
		Gustosa	35.5 ± 2.3	36.0 ± 0.6	+1	ns
	Abaxial	Marian	29.4 ± 1.8	34.4 ± 0.7	+3	*
		Gustosa	36.4 ± 1.3	38.0 ± 0.4	+2	ns
well-watered ➔ water-deficit	Adaxial	Marian	32.5 ± 0.9	27.7 ± 1.1	−8	*
		Gustosa	37.1 ± 3.5	31.2 ± 2.6	−16	ns
	Abaxial	Marian	31.2 ± 1.8	29.7 ± 2.4	−9	ns
		Gustosa	39.1 ± 3.7	32.4 ± 1.6	−17	*

## Discussion

Stomata have a pivotal role in controlling the exchange of CO_2_, O_2_, and water vapor between the plant and the surrounding atmosphere. Stomatal development and functioning are affected by environmental conditions, including light, temperature, CO_2_ concentration, humidity, and soil water availability [4, 10, 12, 13, 32]. As water availability and uptake by plants are essential to plant growth and survival, reductions in stomatal opening in response to water-deficit conditions will limit photosynthesis and affect water-use efficiency. Closing and opening stomata allow plants to react to fluctuating water availability in the short term. More long-lasting stomatal responses to water deficit are found in stomatal development. In line with earlier reports, we found an increase in *SD* and a decrease in *SL* under water deficit as stomatal acclimations in response to drought [12, 13, 40]. While previous studies have shown the acclimatory response of stomata to drought stress, we also investigated the differences in the response of adaxial and abaxial stomata to drought. Richardson et al. (2017) reported that amphistomatic leaves show differential stomatal opening responses between the two surfaces. In this study on basil plants as a model for amphistomatic plants, we investigated if this differential stomatal response also shows in stomatal development, more particularly as a response to drought stress, and came to the following conclusions:

### Water deficit reduces the stomatal size and increases the density both at the adaxial and abaxial leaf surface

We found that leaf stomatal length *SL* decreased due to drought, while stomatal density *SD* increased. Moreover, this response was congruent for both the adaxial and abaxial leaf sides. Epidermal cell area was significantly increased under well-watered conditions. Increased epidermal cell expansion under well-watered conditions decreased stomatal densities. Stomatal index *SI* did not significantly differ between drought treatments (Figures 1E and 1.F), indicating that the difference in *SD* is the result of cell expansion under well-watered conditions [41], as confirmed by the differences in the epidermal cell area. The reduction in leaf size under water-deficit conditions (Figure 3) led to higher epidermal cell density. In combination with the smaller stomata at higher numbers under drought stress, this results in unchanged stomatal indices compared to the well-watered treatment. Similar results were found by Clauw *et al.* (2015) on six *Arabidopsis thaliana* accessions originating from different geographic regions, exposing them to mild drought stress [38].

This difference in *SD* and *SL* between the water-deficit and well-watered treatment was visible at all leaf levels, but not always significant. While early reports showed this increase in *SD* and decrease in *SL* under water deficit on abaxial leaf surfaces, indicating this stomatal acclimation to drought [12, 42], our results show that this acclimatory developmental reaction occurs similarly on both leaf surfaces of amphistomatic leaves of basil. The simultaneous changes in *SL* and *SD* on the adaxial and abaxial leaf sides under water-deficit conditions clearly illustrate the need to study both leaf sides when working on stomata. Although plants can respond to drought stress by reducing the stomatal opening, limiting photosynthesis, and affecting WUE, this is only a short-term response enabling plants to cope with fluctuating water availability. A long-term strategy for persistent water deficit thus involves acclimation of their stomatal development by a reduction in *SL* while increasing *SD*, regulating water loss during these prolonged periods of drought stress.

In dicot plants, cotyledons and leaves have different origins, with leaves formed from vegetative meristems during seedling growth while cotyledons originate as the seeds develop during the embryogenesis process [43]. Cotyledons, being capable of storing nutrients and performing important photosynthetic functioning (for epigeal germinators), provide the necessary matter needed before the development of the first true leaf [44]. Despite the different origin and functioning of cotyledons compared to leaves, their stomatal developmental reaction to water deficit was found to be similar. This emphasizes the fact that already early on in the plant’s development the soil moisture content is registered by the developing plant and this signal is passed on to the growing parts of the plant. To our knowledge, this study is the first to report and highlight this stomatal developmental reaction to water deficit conditions, already starting from the cotyledons and continuing in the leaves. This supports the hypothesis of internal communication between plant parts to create a functional plant able to grow and survive at the location where the seed has germinated.

### Leaves with small stomata in high densities obtain the highest stomatal conductance

Previous studies have shown a negative relationship between stomatal size and density [5, 32]. Our study on basil plants confirms that this relationship exists amongst all leaf levels and both leaf sides combined, described by log-normal equations of *SL* and *SD*, for water-deficit and well-watered treatments separately. This functional trade-off greatly influences *g*_s,cal_, the calculated potential leaf stomatal conductance. Forming leaves with high stomatal densities and smaller stomatal dimensions is therefore an interesting strategy to obtain the highest *g*_s,cal_ values [32]. Small stomata in high densities, linked to low availability of water during cultivation, allow for higher transpiration rates. However, by closing the stomata under water-deficit conditions (Figure 4) the absolute transpiration values were reduced compared to the well-watered conditions. This was shown in Figure 4, where the number of open stomata was used for the calculations of *g*_s,open_. Even though plants grown under water deficit have the potential to transpire more based on their stomatal development, with higher densities of small stomata, not all stomata are opened during drought stress. This increased control resulted in a higher WUE for the water-deficit treatment, showing that plants exposed to drier growing conditions alter their stomatal traits, both development and opening, to optimize their water use and force the water uptake potential of the roots. These results show that the close correspondence between *SL* and *SD* with water availability is consistent with the requirements to increase *g*_s,cal_ under water-deficit conditions. This suggests that cultivation under lower water availability would result in plants with enhanced plant fitness in a broader range of environments, as the relationship between *SL* and *SD* drives both short-term and long-term acclimation of *g*_s,cal_. Moreover, smaller stomata have shorter response times [45–48], which promote higher water use efficiencies, as confirmed in this study.

### Adaxial stomata are more sensitive to water stress than abaxial stomata

Basil leaves have an amphistomatous nature, which allows them to open and close their stomata on both sides independently. Considering that the adaxial leaf side is more exposed to light, wind, dust, precipitation, and other environmental factors, it makes sense that the stomata on the adaxial side of leaves are inherently different from the abaxial leaf side [9]. It should be noted that adaxial and abaxial stomata respond differently to different environmental factors. Richardson *et al.* (2017) found the adaxial stomata of *Eucalyptus globulus* Labill. leaves closed more in response to higher light intensities (natural and artificial), while Wang *et al.* (2011) reported the stomatal response to monochromatic green light (540 nm). They found that the abaxial stomata of sunflower leaves strongly responded to green light, while the adaxial stomata did not. Additionally, the blue light response (470 nm) was higher in the abaxial stomata when they were directly exposed to the light source. Furthermore, Mott and O’Leary (1984) reported a higher sensitivity of adaxial stomata to water stress compared to abaxial stomata. The sensitivity and response of adaxial and abaxial stomata hence differ for different environmental factors, showing the importance of further investigating the stomatal behaviors of amphistomatous leaves [6, 7, 49].

Apart from these functional differences, there are also morphological differences. The adaxial stomata are adjacent to a more tightly packed palisade mesophyll cell layer, while abaxial stomata are adjacent to a more loosely packed spongy mesophyll cell layer. Their contact with the water supply is therefore inherently different [21]. Although the stomatal developmental reaction to water availability is similar for the two leaf surfaces, the adaxial stomata are more sensitive to water deficit than the abaxial stomata, with less open stomata under water-deficit conditions. Under water-deficit, the calculated stomatal conductance based on the number of open stomata (*g*_s,open_) significantly decreased compared to the potential stomatal conductance *g*_s,cal_ for the adaxial leaf surface, with reductions of 49%, 60%, 61%, and 59% for the cotyledons, LN1, 2 and 3, respectively (Figure 4). This remarkable decrease in stomatal conductance for the upper leaf side when taking the open stomata into account was not as pronounced for the lower leaf side, with reductions of 21%, 8%, 1%, and 17% under water deficit conditions. This might be attributed to the higher boundary layer conductance and thus the better contact of the adaxial leaf surface with the environment. Stomata will close and open due to evaporative demand, with factors enhancing water loss causing stomatal closing. Important factors influencing stomatal conductance include light irradiance, boundary layer conductance, and vapor pressure deficit between the leaf and the atmosphere, determining evaporative demand [7]. The surface boundary layer will afford the lowest conductance in the pathway of water vapor from the evaporating leaf, through the stomata to the atmosphere [9]. It is hypothesized that the sensitivity to environmental cues of the adaxial leaf surface is higher as the boundary layer conductance is higher, consequently increasing the effectiveness of stomatal control. Richardson et al. (2017) found that amphistomatic leaf surfaces of *E. globulus* Labill. regulate their gas exchange independently in response to variations in evaporative demand induced by irradiation. This confirms that the individual leaf surfaces respond differently to environmental cues, showing the independent regulation of stomata on both sides of the leaf and suggesting a degree of hydraulic isolation between the leaf surfaces [7, 50]. Our results are in line with the results reported by Driscoll et al. (2006), who investigated the effect of CO_2_ enrichment on adaxial and abaxial stomatal development in maize leaves. Although maize is a C4 plant while basil is a C3 plant, they also found that the abaxial leaf side had a much higher threshold for stomatal closure in response to high CO_2_ and high light than the adaxial leaf side. These results show the importance of understanding the amphistomatic behavior to water loss and transpiration, which leads to optimization of the water-use-efficiency as it indicates that adaxial stomata are programmed to be a more sensitive indicator for changes in water availability than the abaxial stomata [7, 10]. Additionally to acclimating the stomatal development to available water, amphistomatic plants cope with this stressor by rapidly closing their adaxial stomata when the water supply cannot meet the evaporative demands.

The question remains if it is more favorable to breed for amphistomatous plants compared to hypostomatous plants. While amphistomaty provides the advantage of higher CO_2_ diffusion into the leaf for photosynthesis, it has disadvantages regarding pathogen infection. Increasing the stomatal density by locating stomata on both leaf sides, increases the number of potential infection sites [51]. Taking our data into consideration, cultivating under drier soil moisture conditions, hence decreasing the stomatal length, could lower the incidence of pathogen infection in amphistomatous plants compared to those grown under well-watered conditions. The acclimation advantage of amphistomaty over hypostomaty considers many parameters: high light environments, leaf thickness, available water, and boundary layer resistance [7, 16, 18, 19]. This means that fast-growing plants, such as herbaceous plants, and those growing in high-light environments benefit from amphistomaty [7, 51]. Muir (2018) investigated the influence of light and growth form on the stomatal ratio among British angiosperms. He investigated under what ecological conditions CO_2_ limited the photosynthetic rate, as he hypothesized that amphistomaty is evolutionarily favored when CO_2_ limits the photosynthetic rate. Under high light, CO_2_ can strongly limit photosynthetic rates, thus by adding a parallel pathway for CO_2_ diffusion by putting stomata on both leaf surfaces, this limitation is relieved. He does emphasize that this benefit of amphistomaty works better in coordination with higher stomatal density [16]. In conclusion, breeding crops with a certain stomatal ratio (ranging from hypostomatous to amphistomatous) depends on the targeted plant traits and environmental conditions: fast-growing versus the higher incidence of pathogen infection, high light environment versus moderate light environment, and thick leaves versus thin leaves. Further research is required to fully understand the influence of the stomatal ratio on plant growth, quality, and resilience.

### The acclimation of stomatal development to water availability opens possibilities to increase water-use efficiency without sacrificing biomass production

Both abaxial and adaxial leaf sides responded in a similar way to changes in soil moisture content. Plants that were transferred from well-watered to water-deficit conditions developed leaves with a higher stomatal density, while reducing their stomatal size (Table 2) compared to the control plants grown entirely at well-watered conditions. Reversing the soil moisture content, going from water-deficit conditions to well-watered resulted in a reversal of the stomatal developmental changes: a reduction in stomatal density while increasing stomatal length. We also observed that plants cultivated under water deficit, having more but smaller stomata, showed high WUE, compliant with the results of Xu and Zhou (2008). Furthermore, exposing plants to water-deficit conditions and then switching them to well-watered conditions showed a significant increase in biomass production while maintaining a high WUE (Figure 7). However, it can be questioned how long this high WUE would be maintained as the new leaves acclimate to the well-watered soil conditions. The newest leaves produced will be less efficient with larger stomata at lower densities. This means that their old leaves can better withstand drought stress than their new leaves which developed under well-watered conditions. In the context of efficient use of resources and obtaining plant production with high yield, these results are of great value. The design of a strong evaporative plant, with small, fast, and responsive stomata by irrigating less in the early stage of the cultivation period results in plants with high WUE. The reduced biomass production can then at a later stage in the cultivation period be compensated by increasing the irrigation, as shown by our results where the biomass production recovered when switching to well-watered conditions (Figure 7). Improving the water use efficiency is desirable if biomass yield can still be maintained. The acclimation of stomatal development to water availability opens possibilities to increase water-use efficiency with minimally sacrificing biomass production.

## Conclusions and perspectives

We have shown that basil plants acclimate the stomatal density and size of the newly formed leaves to the water availability in the soil. This acclimation was shown to result in better control of the leaf conductivity for plants grown under water-deficit conditions. As water scarcity is challenging current plant production systems, this finding is of great significance for agricultural crop production. The increasing demand of consumers for high-quality and sustainable plant production forces growers to optimize their irrigation systems or evolve to more high-tech systems such as vertical farms. By growing plants close to water deficit, they can be triggered to invest in a better-developed transpiration system resulting in stems and leaves which can cope better with variations in water availability. Our findings illustrate that in addition to stem architecture, water deficit also influences stomatal development, resulting in plants with a high WUE and *g*_s,cal_. Exposure to continuous water deficit does not only influence stomatal development, plants also sense and communicate the current water status to developing leaves to induce an appropriate developmental response that optimizes their resource use.

This research is the first to provide evidence that abaxial and adaxial stomata respond similarly to water deficit. Although amphistomatic leaf surfaces regulate gas exchange independently in response to variations in evaporative demand, this study proves that on a stomatal developmental level, their response is identical. Both leaf surfaces increase their stomatal density while decreasing stomatal length when exposed to water deficit. However, adaxial stomata are more sensitive to water deficit, closing their stomata more and sooner compared to abaxial stomata.

From the observed responses of stomata to short-term environmental changes, influencing their ability to withstand fluctuating environmental conditions in the longer term, it can be concluded that stomata are key tools for a plant to optimize its water use efficiency. This opens possibilities to grow plants with optimal stomatal characteristics to withstand and anticipate future environmental changes. Further molecular work is recommended to unravel the underlying mechanisms which regulate this stomatal developmental response to water deficit.

## Materials and methods

### Plant material and growth conditions

Basil plants grown under water-deficit conditions were compared to plants grown under well-watered conditions. In a follow-up experiment, the water availability was switched to investigate the acclimation of the newly developing leaves to the current water status. Experiments were carried out in 2 to 4 growth chambers (Duffel, Belgium). Two basil varieties were used, both Genovese cultivars: *O. basilicum* L. cv. Gustosa and *O. basilicum* L. cv. Marian (Enza Zaden, Enkhuizen, The Netherlands). Seeds were planted in plastic containers (230 cm^3^) with a substrate containing a mix of peat (^2^/_3_), compost (^1^/_6_), and perlite (^1^/_6_). The sown plant containers were kept in a germination room for three days at 92 ± 1% RH and 21.2 ± 1.5°C in total darkness. Three days after sowing (3 DAS, *Days After Sowing*), they were transferred to growth chambers of 2 m^2^, each containing 70 plant containers. Irrigation was set as previously reported by Driesen et al. (2021). Briefly, when the substrate volumetric water content decreased below a calculated threshold, ebb and flow irrigation was applied for 4 minutes. Six containers per treatment were equipped with soil moisture sensors (Soil Pro Mini, Sigrow BV, Wageningen, The Netherlands). Soil moisture treatments were initiated immediately after placing the containers in the growth chambers. The electrical conductivity (EC) of the nutrient solution (Kristalon Label Blue, YaraTera, Vlaardingen, The Netherlands) was ~0.7 mS cm^−1^ at 21°C. Each growth chamber contained 2 LED lamps, supplying a broad white-light spectrum to the plants. The photoperiod was set at 16 hours, from 6:00 a.m. until 10:00 p.m. resulting in a daily photosynthetically active radiation (*PAR*) of approximately 170 μmol m^−2^ s^−1^ at plant height. The temperature was 20.5 ± 2.7°C and the relative humidity was 77 ± 6%. The experiments were repeated twice (December 2021 until April 2022). At the end of the cultivation period, each basil plant contained cotyledons and 3 leaf levels, each leaf level containing 1 leaf pair. Plant density (the number of basil plants per plant container) equals 13 ± 2 plants per container.

### Substrate matric potential during cultivation

A substrate-specific water retention curve was established for the substrate used to link the corresponding pF (*p* of *“Potenz”, F* of *“Freier Energy”*, translated to “exponentiation of free energy”) values to the substrate VWC (*volumetric water content*). To estimate the substrate-specific pF curve, samples were first taken from the substrate using Kopecky rings (stainless steel soil sample rings). The ring was pressed into the substrate and the remaining substrate around the ring was removed. Subsequently, these samples were saturated and brought into equilibrium step by step with increasing moisture tension values, pF values, for 7 days each time. As a result, water was gradually withdrawn from the substrate samples. A sandbox (pF 0–2), low-pressure boilers (pF 2.3 and 2.8), and high-pressure boilers (pF 3.4 and 4.2) were successively used. After each equilibration, the sample was weighed and finally dried in a drying oven at 70°C for a period of 7 days. The matric potential is translated into a height *h* (in cm). The decimal logarithm of this height (^10^log(−*h*)) is called the *pF* value or moisture tension. The pF value is plotted against the volumetric moisture content (cm^3^ of water per cm^3^ of soil).

VWC values of 20% and 50% were found to correspond to pF values of 1.95 and 1.15. With pF 2 being the theoretical wilting point (−10 kPa) and pF 1 being the container capacity (−1 kPa) [28], the VWC of the two treatments oscillated respectively around these critical pF values. During container cultivation, substrate water is generally considered available for plants between matric potentials of −1 to −10 kPa [29, 30]. For clarity, the two treatments will be named as follows: water-deficit treatment or “pF1.95” and well-watered treatment or “pF1.15”. The water content [%] of the leaves per plant container was calculated to check the leaf water status. The water content in the plant’s leaves grown under water-deficit conditions (91.4 ± 0.8%) was significantly lower than in the plants grown under well-watered conditions (92.6 ± 0.7%). This supports the lower water availability under water-deficit conditions, analyzed using a non-parametric Wilcoxon rank sum test (*n* = 6).

Additionally, to explore the acclimation of leaf structure and stomatal anatomy to water availability further, plants were either grown at water potentials of pF1.95 (water-deficit) or pF1.15 (well-watered) up to 35 DAS, the point where LN3 (LN, *leaf number*) became visible. Half of the plants were then switched to the other water potential (pF 1.15 and pF1.95 resp.) and all plants were grown for another 7 days until LN3 of all plants had reached maturity with a leaf surface of minimum 20 cm^2^. At this developmental stage, epidermal peels (see further under “Light microscopy”) of the abaxial and adaxial leaf sides of 6 leaves per treatment were taken. Leaves of 42 DAS plants kept at the same matric potential (“No VWC change”) were compared to leaves of plants that had been changed on 35 DAS to another matric potential (“After VWC change”). This experiment was done twice, the first time with basil cv. Marian, and the second time with cv. Gustosa.

### Light microscopy

To quantify the leaf structure and stomatal anatomy, epidermal tissue was stripped from the adaxial and abaxial surfaces of basil leaves at every leaf level. For this, the leaf surface was coated with clear nail polish in an area between the central vein and the leaf edge, leaving it to dry for 5 minutes. Clear tape was attached on top of the dried nail polish, allowing it to peel off the epidermal tissue imprint from the leaf surface and place it on a glass slide [12, 31]. To quantify the stomatal (*SD*) and epidermal (*ED*) densities, the cell numbers were manually counted for each independent sample (0.55 mm^2^ sample surface) using an Olympus BX40 microscope (20X objective magnification). Measurements of stomatal size (length and width), stomatal pore area, and epidermal cell area were manually determined using the software program ToupView (ToupTek, 2020). Stomatal size *SL* was determined as the length between the connections of the guard cells at each end of the stoma [12]. The stomatal index was calculated using the formula: [*SD*/(*ED* + *SD*)] x 100. Imprints were taken at 19, 24, 27, 32, and 35 DAS both abaxial and adaxial for six leaves from all leaf numbers and for each treatment.

### Stomatal conductance calculations and measurements

Calculations of the potential stomatal conductance were made to enable a direct interpretation of the effect of water availability on the transpiration potential resulting from the anatomical features of stomata. To this end, stomatal size and density parameters were used to calculate the potential stomatal conductance *g*_s,cal_ [mol m^−2^ s^−1^] as follows [32]:

(1) \begin{equation*} {g}_{s, cal}=\frac{\frac{d}{v}\ x\ SD\ x\ {a}_{max}}{l+\frac{\pi }{2}\ \sqrt{\frac{a_{max}}{\pi }}} \end{equation*}

Where *d* is the diffusivity of water in air [m^2^ s^−1^], taken as equal to 2.2386 x 10^−5^ m^2^ s^−1^ at the treatment level of 24°C. *SD* is the stomatal density [mm^−2^] and *a*_max_ is the average stomatal pore size [μm^2^]; *v* is the molar volume of air at 24°C [m^3^ mol^−1^] and was set at 24.383 m^3^ mol^−1^ for all treatments. The stomatal pore depth *l* was calculated as half the guard cell width, assuming that guard cells inflate to a circular cross-section [32]. The stomatal anatomical parameters *a_max_*, *SD* and *l* determine potential stomatal conductance to water vapor. An increase in stomatal density (*SD*) impacts *g*_s,cal_ positively. On the one hand, smaller stomata will result in a higher *g*_s,cal_ compared to larger stomata, because *g*_s,cal_ is inversely proportional to the distance that gas molecules have to diffuse through the stomatal pore (*l*). This stomatal pore depth (*l*) increases with stomatal size *SL* because guard cells inflate circularly in cross-section. On the other hand, longer and wider stomata, denoted by *a*_max_, promote gas exchange [15, 32]. The potential stomatal conductance *g*_s,cal_ was calculated separately for each leaf side and each leaf number.

Leaf stomatal conductance (*g_s_*, in mmol m^−2^ s^−1^) was measured in addition using an SC-1 Porometer connected to a microcontroller (Decagon Devices, METER group, Washington, USA). These measurements were conducted twice in the second experimental replication (cv. Gustosa), at 35 DAS (before VWC change) and 42 DAS (after VWC change). Fifteen (35 DAS) and five (42 DAS) plants for each treatment were selected from different plant containers. Stomatal conductance was measured abaxially and adaxially on opposite leaves on LN2. The stomatal conductance of the total leaf area of these LN2 leaves [mol s^−1^] was consequently calculated using the measured *g_s_* [mmol m^−2^ s^−1^], multiplying this value with the total leaf area [cm^2^] of LN2 leaves. To calculate the leaf area, we assumed the shape of basil leaves as a diamond, thus multiplying the values of leaf length [cm] and leaf width [cm], and dividing by 2. Correction of units is done by dividing the final values by 10 to display the stomatal conductance of the total leaf area of LN2 in mol s^−1^. Subsequently, epidermal peels were made of these abaxial and adaxial sections to determine their stomatal anatomy.

### Biometric measurements

At 35 DAS, the morphological traits of the plants were measured. The fresh weight of the stems and leaves per plant container was measured separately for 6 containers per treatment. Water Use Efficiency (WUE, in g L^−1^) was calculated as the ratio between the fresh weight of leaves and stems [g] and the amount of water transpired [L], calculated by the use of the soil moisture sensors as described by Driesen et al. (2021). Briefly, the VWC measured by the sensors was converted to mL container^−1^ using a substrate-specific equation. By subtracting the VWC before irrigation (minimum VWC at irrigation moment *n* + 1) from the VWC after irrigation (maximum VWC at irrigation moment *n*), daily evapotranspiration [mL day^−1^ container^−1^] was calculated. By integrating the daily evapotranspiration over the cultivation period, the total evapotranspiration [mL container^−1^] was calculated [3]. This total evapotranspiration sum is the amount of water transpired [L] per plant container.

### Statistical analyses

The univariate normality of the measured parameters was tested using the Shapiro–Wilk test. An ANOVA test was used to compare the average values of the different treatments (p < 0.05) if the data was normally distributed. The non-parametric Wilcoxon rank sum test was used when the data was not normally distributed. Statistical analyses were performed using JMP Pro 16 (SAS Institute INC., Cary NC, USA) and R Studio (version 4.0.3). Prism (GraphPad Software) was used to generate the graphical representations of the data.

## Data Availability

All data can be obtained from the corresponding author upon request.
